# PTIP-Associated Protein 1: More Than a Component of the MLL3/4 Complex

**DOI:** 10.3389/fgene.2022.889109

**Published:** 2022-06-09

**Authors:** Bo Liu, Zhen Li

**Affiliations:** Department of Human Anatomy, Histology and Embryology, the Fourth Military Medical University, Xi’an, China

**Keywords:** PA1, histone lysine methylation, adipogenesis, humoral immunity, steroid receptor, spermatogenesis, embryonic development

## Abstract

PTIP-associated protein 1 (PA1) is a unique component of MLL3/4 complexes, which are important mammalian histone 3 lysine 4 (H3K4) methyltransferases. PA1 has generated research interest due to its involvement in many essential biological processes such as adipogenesis, B cell class switch recombination, spermatogenesis, and embryonic development. In addition to the classical role of PA1 in H3K4 methylation, non-classical functions have also been discovered in recent studies. In this review, we systematically summarize the expression pattern of PA1 protein in humans and sort the specific molecular mechanism of PA1 in various biological processes. Meanwhile, we provide some new perspectives on the role of PA1 for future studies. A comprehensive understanding of the biological functions and molecular mechanisms of PA1 will facilitate the investigation of its complicated roles in transcriptional regulation.

## Introduction

Methylation modification on histone lysine residuals is linked to a wide variety of essential cellular processes such as transcription and DNA repair (Audia and Campbell, 2016; Husmann and Gozani, 2019; Jambhekar et al., 2019). One of the most intensely studied histone modifications, histone 3 lysine 4 methylation (H3K4me), which is catalyzed by histone methyltransferases, has been widely verified to promote gene transcription (Park et al., 2020). Kmt2 family, known as mammalian H3K4 methyltransferases, is mostly classified as MLL1/2, MLL3/4, and SET1A/B (Husmann and Gozani, 2019). As a scaffold, these enzymes recruit some other proteins constituting large complexes to perform their functions. While the WRAD subcomplex comprised of ASH2, hDPY30, RBBP5, and WDR5 exist as a common component in the KMT2 complexes, each complex also has its exclusive constituents such as MENIN in MLL1/2 complexes, WDR82 and CXXC1 in SET1A/B complex, and PTIP, PTIP-associated protein 1 (PA1), and UTX specifically existing within MLL3/4 complexes (Froimchuk et al., 2017).

**FIGURE 1:**
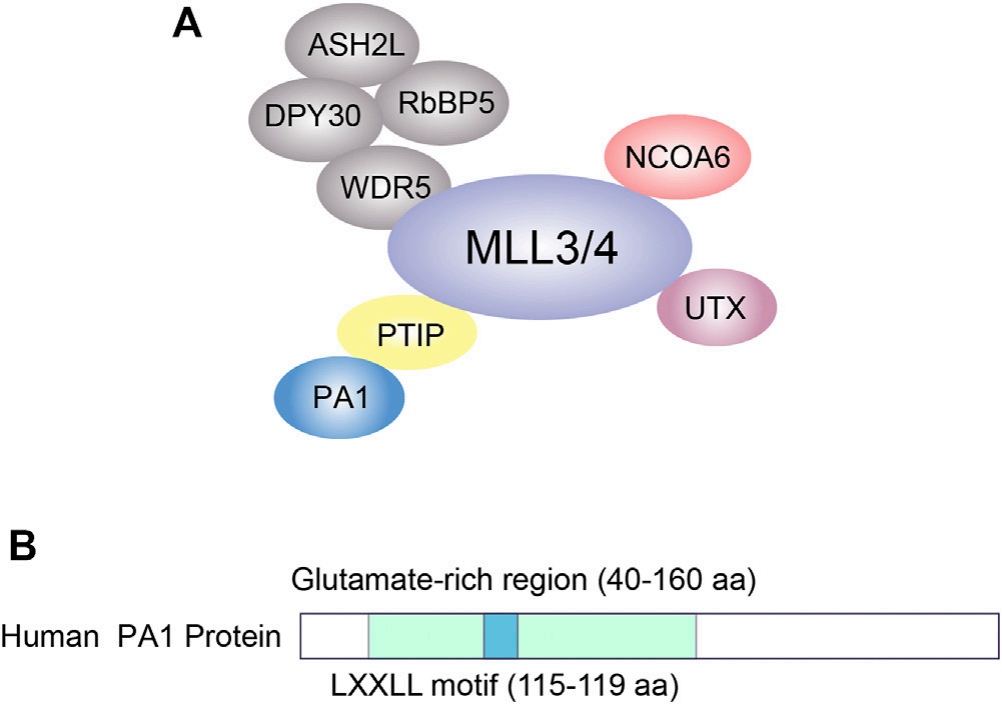
The schematic image of KMT2C/D complex **(A)** and the protein structure of PA1 protein **(B)**.

As one of the unique components of MLL3/4 complexes, PA1 was primarily found to interact with PTIP within the MLL3/4 complexes (Figure 1A) (Cho et al., 2007). The human PA1 protein is composed of 254 amino acids (Figure 1B) while the mouse PA1 protein consists of 253 ones, and the identity of the amino acid sequences between these two species is up to 87.4%. The 47–160 aa constitute a central glutamate-rich region within the human PA1 protein while the LXXLL motif located at the 115–119 aa has been proved to be responsible for binding with steroid receptor coactivator 1 (Figure 1B). PA1 is ubiquitously expressed in different human organs though the abundance of PA1 protein in different organs and tissues varies (Liang et al., 2009). According to the GeneCards database, the mRNA of human *PA1* is highly expressed in the cerebellum while the protein of PA1 was abundant in the B lymphocyte, fetal gut, testis, and CD8^+^ T cells, indicating the multiple potential functions of PA1 in different organs (Supplementary Figure S1) (Fishilevich et al., 2016; Stelzer et al., 2016).

**FIGURE 2:**
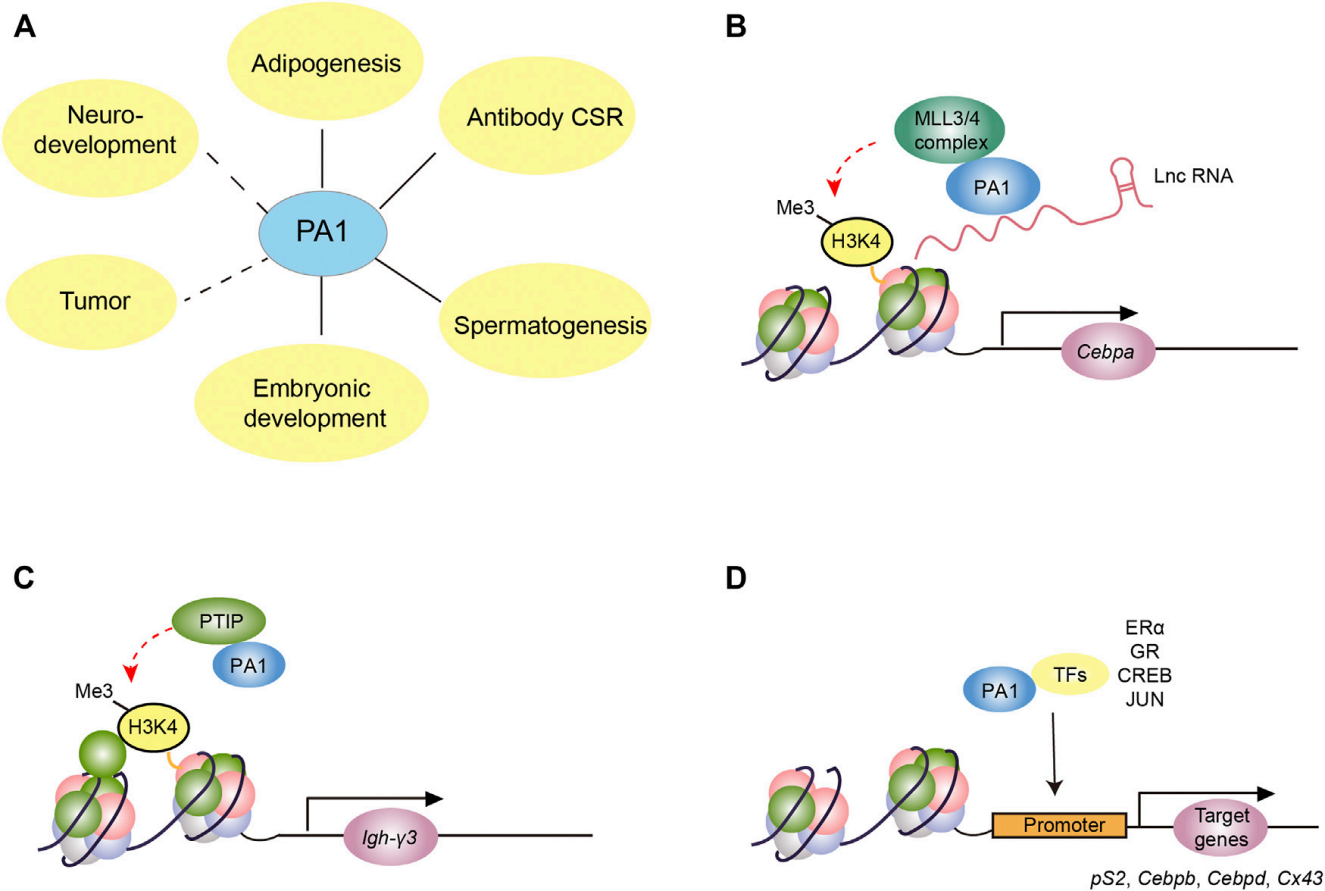
The molecular function of PA1 in different biological processes. **(A)**The involvement of PA1 in different biological processes. The solid line indicates the verified relationship between PA1 and the biological process while the dashed line denotes the potential functions of PA1 in these biological processes. **(B)** PA1 functions as a link between lncRNA and MLL3/4 complex and participates in MLL3/4-dependent H3K4me in promoting the expression of CEBPA in adipogenesis. **(C)** PA1, together with PTIP, is involved in the transcription initiation of noncoding germline transcripts such as Igh-γ3 through MLL3/4-independent H3K4me in B cell CSR. **(D)** PA1 binds with some transcription factors including steroid receptors, SMADs, CREB, and JUN to regulate the downstream gene expression such as *Ps2, Cebpd,* and *Cx43*.

PA1 has been validated to participate in versatile biological processes such as adipose differentiation (Lee et al., 2020; Xiao et al., 2015; Starnes et al., 2016), B cell class switch recombination (CSR) (Starnes et al., 2016), spermatogenesis (Liu et al., 2022), embryonic development (Takeshita et al., 2013; Kumar et al., 2014; Takada et al., 2017), tumor process (Takeshita et al., 2013; Kumar et al., 2014; Takada et al., 2017), and neurodevelopment (Daum et al., 2021) (Figure 2A). As PA1 was first identified as a unique component of the MLL3/4 complex, its specific roles in H3K4 methylation are considered to be the classical functions (Figures 2B, C). In addition, some non-classical functions of PA1 such as interacting with different types of steroid receptors and other transcription factors have also been reported (Figure 2D) (Liang et al., 2009; Zhang et al., 2013). In this review, we systemically summarize the established biological functions of PA1 and discuss its potential functions, in an attempt to provide new insights into the unknown roles of PA1 for further exploration.

## Classical Function of PA1: Participating in H3K4 Methylation

### MLL3/4-Dependent H3K4ME Modification

Abnormal adipose differentiation and maturation could lead to body mass variations such as obesity or low body weight and even other severe adipogenic differentiation-related disorders (Hammarstedt et al., 2018; Ghaben and Scherer, 2019). In the complex network of transcriptional regulation of adipogenesis, PPARγ and C/EBPα are considered the most important transcription factors (Lee et al., 2019). Recent studies have shown that PA1 could interact with adipogenic differentiation induced noncoding RNA (ADINR) mediating the MLL3/4 complexes to localize the promoter of *Cebpa* so as to elevate the H3K4me3 level on its promoter; thus, enhancing the expression of C/EBPα and promoting adipose differentiation and maturation (Xiao et al., 2015) (Figure 2B). RNA pull-down experiment has validated the direct interaction between PA1 and ADINR. By constructing various truncated ADINRs, it was found that the 1,685–1,937 nucleotides, a region that includes a LINE repeat element conserved in mammals, were indispensable for ADINR to bind with PA1 (Xiao et al., 2015). Furthermore, PA1 ChIP results also demonstrated that PA1 was recruited to the promoter of *Cebpa*, and the absence of ADINR significantly inhibited the binding of PA1 to the promoter of the *Cebpa*. In addition to that, they also detected the increased H3K27me3 level around the promoter of *Cebpa* after knocking down the ADINR, probably due to the absence of the H3K27me2/3 demethylase UTX of MLL3/4 complexes. This novel research suggested that PA1 functions as the link in *cis* lncRNA ADINR recruiting MLL3/4 complexes to affect the expression of C/EBPα (Xiao et al., 2015). This is the first finding that evidenced the important role of PA1 in the MLL3/4 complex. More importantly, it showed its potential in identifying whether other lncRNAs containing a LINE repeat element could also interact with PA1 in regulating H3K4me modification.

### MLL3/4-Independent H3K4ME Modification

In contrast to their effects on adipose differentiation, PA1 was necessary for the transcription initiation of noncoding germline transcripts in B cell CSR while the MLL3/4 complexes are indispensable in this critical process (Starnes et al., 2016). Accurate coordination of B cell immunoglobulin heavy chain (IgH) CSR is essential to the proper adaptive immune response and maintaining the stability of the B cell genome (Methot and Di Noia, 2017; Yu and Lieber, 2019). The initial step of CSR is the transcription of the switch region in the genome which encodes noncoding germline transcripts so as to recruit the activation-induced cytidine deaminase (AID) to produce double-strand breaks and subsequently induce the generation of diverse types of immunoglobulins (Alt et al., 2013; Stavnezer and Schrader, 2014). Linda M. Starnes *et al.* found that in the B cell-specific *Pa1* knockout mouse, the transcription of Igh-γ3 in the S region was defective and the expression of mature IgG3 was significantly decreased (Starnes et al., 2016) (Figure 2C). Meanwhile, the H3K4me3 signals at Igh-γ3 and Igh-γ2b sites decreased significantly in these *Pa1* knockout B cells, suggesting that PA1 may promote CSR by upregulating the H3K4me3 modification. During this process, the knockout of *Ptip* in B cell presented a similar phenotype compared with *Pa1* knockout B cells, implying the close relationship of PA1 and PTIP in B cell CSR. However, PA1 seems to be independent of MLL3/4 complexes in promoting transcription during this process. When the BRCA1 C-terminus 3–6 (BRCT 3–6) domain within PTIP which is required for the interaction of PA1 and PTIP with MLL3/4 was deleted, the CSR process was not affected (Starnes et al., 2016). This phenomenon is inconsistent with the previous view that the PA1 and PTIP participate in H3K4 methylation through MLL3/4 complexes, and therefore, whether the PA1-PTIP subcomplex regulates H3K4me3 modification by mediating other histone methyltransferase complexes remains to be further elucidated.

Participation in histone lysine methylation is considered the classical function of PA1. However, its intrinsic mechanism seems more complicated than previously thought. On one hand, it remains undetermined whether PA1 functions as a link in the localization of MLL3/4 complexes to further catalyze H3K4me3 and H3K27me3 at specific locus or whether there exist other lncRNAs interacting with PA1 during other important cellular processes. On the other hand, how the PA1-PTIP subcomplex performs histone methylation independent of MLL3/4 complexes in B cell CSR still remains unclear.

## Non-Classical Function of PA1: Interacting With the Transcription Factors

The non-classical functions of PA1 lie in its close and extensive interaction with other nuclear transcription factors, including the steroid receptors, phosphorated CREB, and JUN.

### Regulating the Activity of Steroid Receptors

Steroid receptors, as ligand-activated transcription factors, have been reported to be pivotal to development, tumorigenesis, reproduction, and other processes (Levin and Hammes, 2016; Truong and Lange, 2018; Skowron et al., 2019). Some studies reported that PA1 regulated the intrinsic transcriptional activity of steroid receptors despite the effect and extent of PA1 regulation on steroid receptors varies (Liang et al., 2009; Zhang et al., 2013). The first discovered steroid receptor which could be regulated by PA1 is the canonical estrogen receptor α (ERα) (Liang et al., 2009). PA1 was found to interact with steroid receptor coactivator 1 (SRC1), a coactivator of ERα (Liang et al., 2009; Glass and Rosenfeld, 2000). The LXXLL motif (L for leucine and X for any possible amino acid) in PA1 was responsible for binding to SRC1, and its C-terminal is required to bind with the N-terminal of ERα (Figure 2D). PA1 could promote the transcription of the downstream genes of ERα, and it was speculated that its central glutamate-rich region (poly-Q) was responsible for this effect. ChIP results showed that PA1 is localized at the promoter region of *pS2*, a classical target gene of ERα. Nevertheless, the H3K4me3 level of the promoter region of *pS2* was not affected after knocking down the *Pa1*, implicating that the regulation of PA1 on the ER targeted genes did not depend on the classical histone methylation (Glass and Rosenfeld, 2000; Liang et al., 2009). Flow cytometry assay found that *Pa1* knockdown inhibited the transition of MCF-7 cells from the G1 phase to the S phase during mitosis, implying the potential function of PA1 in cell proliferation in ER^+^ breast cancer.

In contrast, PA1 inhibits the transcriptional activity of glucocorticoid receptor (GR) (Zhang et al., 2013), which is imperative in normal development, differentiation, metabolism, neural activity, and homeostasis (Madalena and Lerch, 2017; Majer-Lobodzinska and Adamiec-Mroczek, 2017; Vitellius et al., 2018). By constructing various truncated GRs, it was found that the DNA binding domain of GR is indispensable for the inhibitory effect of PA1. Studies on mechanisms demonstrated that PA1 negatively regulates GR by inhibiting the binding of GR to the glucocorticoid response element (GRE) or by inhibiting its intrinsic activity through forming a PA1-GR-GRE complex. For example, for *Ip6k3*, PA1 inhibits the binding of GR and GRE, while for *Igfbp1*, PA1 functions through these two aforementioned mechanisms (Zhang et al., 2013). In addition, it was also validated that PA1 could bind to AR and inhibit the transcriptional activity of AR (Zhang et al., 2013).

### Regulating the Activity of Other Transcription Factors

Recently, PA1 was reported to be indispensable for the brown adipose tissue (BAT) and muscle development (Lee et al., 2020). The specific deletion of *Pa1* in the precursor cell which could develop into BAT and skeletal muscle cells in the back would cause the death of newborn pups due to defective breath muscles. Further investigation showed that in the primary preadipocytes, PA1 could interact with phosphorated CREB and ligand-activated GR to induce the expression of C/EBPβ and C/EBPδ, which are pivotal transcription factors during the early phase of adipose differentiation. Also, the induction of PA1 on C/EBPβ was found independent of the MLL3/4 complex, suggesting the non-canonical function of PA1 protein in this process (Figure 2D). Given the classical function of PA1 in anchoring the MLL3/4 complex to the promoter of *Cebpa*, we could conclude that PA1 is a key factor in adipose differentiation (Xiao et al., 2015; Lee et al., 2020).

In addition to its role in nuclear receptor activity, PA1 also interacts with SMADs, which are known for their effects on the TGF-β/BMPs pathway (Baas et al., 2018). Previous studies have found that SMADs bind with histone acetyltransferases, such as p300 and the PCAF, and interact with PTIP (Van Nuland et al., 2013; Janknecht et al., 1998). Different activating signaling ligands could activate different SMAD proteins. BMPs trigger the phosphorylation of SMAD1/5/9, while TGF-β causes phosphorylation of SMAD2/3. The SMAD6 and SMAD7 function as negative regulators in this pathway. PA1 has been confirmed to be involved in TGF-β responsive gene activation. While TGF-β SMADs but not the BMP SMADs could interact directly with PTIP and PA1 (Baas et al., 2018) (Figure 2D). *Pa1* knockdown or *Ptip* knockdown in U-2 OS cells both could cause defective expression of TGF-β responsive genes instead of BMP responsive genes (Baas et al., 2018).

Our latest published work also validated the vital role of PA1 in mouse spermatogenesis (Liu et al., 2022). Spermatogenesis is a highly specialized process, subject to the precise internal differentiation of spermatogenic cells and external direct or indirect regulation from Sertoli cells, Leydig cells, or other cells (Griswold, 2016). We found that PA1 was abundant in mice testis and mainly localized at the nuclei of human and mouse Sertoli cells. The specific knockout of *Pa1* in mice Sertoli cells led to the destruction of the blood–testis barrier and aberrant spermiogenesis, contributing to the failure of spermatogenesis. Further transcriptome and Cut-Tag results revealed a subset of genes regulated by PA1 and identified some potential transcription factors which may cooperate with PA1 in this process. Indeed, PA1 could interact with the known AP-1 transcription factor, JUN, and co-regulating the transcription of *Cx43*, which was proven to be required for the Sertoli cell in maintaining proper spermatogenesis (Figure 2D). Moreover, PA1 signals were also detected in mouse Leydig cells, spermatogonia, and spermatocytes, implicating its potential functions in these cells. As such, further investigation of PA1 in these cells is required to fully probe the role of PA1 in spermatogenesis.

## Other Functions of PA1: The Mechanism Remains to be Elucidated

### PA1 is Indispensable for Embryonic Development

PA1 has been proved to play an important role in the development of ectoderm (Kumar et al., 2014). The *Pa1* knockout mouse embryos presented severe developmental defects that occurred in E8.0 and no homozygous mutant could survive to E10.5. During mouse embryonic development, PA1 was mainly expressed in the ectoderm and villous ectoderm in pre-gastrula and was upregulated in the embryonic body after gastrula formation. The *Pa1* ^*−/−*^ embryos can successfully have the anterior and posterior axes established, and the structures of neuroectoderm, mesoderm, and endoderm were normally arranged, yet the embryonic development was stagnated at the stage of 4 and 5 segments and underwent no axial rotation (Kumar et al., 2014). *Pa1* ^*−/−*^embryos had major defects in the abnormal development of extraembryonic tissues and in the development of amnion, chorion, and visceral yolk sac. At the molecular level, the expression of bone morphogenetic protein 2 (BMP2) which is an important factor for extraembryonic development in *Pa1* ^*−/−*^ embryos was significantly downregulated (Gavrilov and Lacy, 2013; Rogers et al., 2015). Therefore, it is speculated that the severe defects caused by the deletion of *Pa1* in mice may be partly due to the deficiency of BMP2. However, whether PA1 regulates BMP2 expression through its classical histone methylation function or through regulating the activity of ERα or other unknown mechanisms remains to be further elucidated.

### PA1 Participates in DNA Damage Repair

Exogenous and endogenous stress could lead to DNA damage, which further elicits the cell cycle checkpoint, DNA damage repair, and other responses. Defective DNA damage response subsequently results in genomic instability, or even cell death, as it was validated in various processes such as a Parkinson’s disease, pulmonary arterial hypertension, spermatogenesis, and cancers (Jackson and Bartek, 2009; Wang et al., 2010; Mouw et al., 2017; Wang et al., 2017; Sharma and Aldred, 2020; Gillman et al., 2021; Gonzalez-Hunt and Sanders, 2021). PTIP was found to act downstream of γH2AX-MDC1-RNF8 in the DNA damage signal transduction cascade. During this process, PA1, together with PTIP, colocalized with the γH2AX; thus, forming a stable complex that was required for cell survival after ionized radiation (Gong et al., 2009). Meanwhile, the deficiency of MLL3 did not disturb the recruitment of PA1 to the DSB sites, suggesting the involvement of PA1 is largely independent of MLL complexes during this process. However, it still remains unknown as to why this subcomplex is only required in IR-induced DNA damage but seems dispensable in other chemical-induced damages, as well as the intrinsic mechanism of how PA1 contributes to the subsequent DNA damage repair.

### Prospective Biological Function of PA1 Protein

The potential biological functions of PA1 are worthy of notice. First, PA1 is widely expressed in different tumor cell lines and the intrinsic function requires to be further uncovered (Supplementary Figure S2) (Fishilevich et al., 2016; Stelzer et al., 2016). Several studies have reported that PA1 may be a potential tumor suppressor (Takeshita et al., 2013; Takada et al., 2017). Among them, the nuclear expression of PA1 was found to be an independent prognostic indicator for relapse-free survival (RFS) of breast cancer patients without lymph node metastasis (Takeshita et al., 2013). Mamoru Takada *et al.* found that the RFS and overall survival rate (OS) of breast cancer patients with BRCA1 mutation accompanied with PA1 deficiency were significantly lower than those without PA1 deficiency (Takada et al., 2017). In addition to that, PA1 seems to be related to the tumor suppressor function of the H3K27 demethylase UTX (Kato et al., 2020). *UTX* mutations were identified in many types of human cancers including acute lymphoblastic leukemia, bladder carcinoma, and medulloblastoma (Mar et al., 2012; Robinson et al., 2012; Cancer Genome Atlas Research, 2014). A recently published study validated that one mutant, G137V, compromised the ability of UTX to bind with the MLL3/4 complex components including PA1, PTIP, and ASH2L and presented aberrant cytoplasm localization and a relatively unstable state, leading to the failure of UTX recruitment to the MLL3/4 target genes (Kato et al., 2020). Intriguingly, they also found that the Δ80-397 mutant of UTX presented stronger interaction, especially with PA1 compared with ASH2L, suggesting that the C-terminal of UTX might be responsible for binding with PA1, and the close interaction of PA1 and UTX should be further investigated. Nevertheless, considering its close relationship with ERα which is critical in breast cancer development, PA1 may theoretically promote the progression of breast cancer, yet the underlying mechanism could be far more complex than thought. More evidence is required to examine its precise effect on breast cancer.

Additionally, the human 6 kb *PA1* gene is located on the c16p.11.2 which is one of the most frequent locations of chromosome copy number variations (CNVs) (Jacquemont et al., 2011). The prevalence of 16p11.2 600 kb BP4-BP5 breakpoint (BP) deletions and reciprocal duplications are both up to 1/1,000, and humans with these CNVs may present intellectual disability (ID), autism spectrum disorders (ASD), and abnormal body mass in humans (Bochukova et al., 2010; Walters et al., 2010; Cooper et al., 2011). Some evidence also showed that PA1 might be involved in the aforementioned aberrant neural symptoms (Bochukova et al., 2010; Walters et al., 2010; Cooper et al., 2011). CNVs at the 16p11.2 locus are also related to cognitive deficits and autistic traits. In patients with 16p11.2 CNVs, phonological processing and language disorders account for 56% (deletion) and 46% (duplication), respectively (Hanson et al., 2015). Given the above evidence, more attention should be placed on the function of PA1 in nervous system development.

Recent studies have shed light on the role of PA1 in the development of the human nervous system (Daum et al., 2021). Hagit Daum *et al.* found a homozygous missense variant of the *PA1* gene (c.274A > G; p.Ser92Gly, NM_024516.4) in three cases of Ashkenazi Jews ancestry from two completely unrelated families, exhibiting severe neurodevelopmental disorders including prenatal clinical features of microcephaly, polyhydramnios, severe developmental delay, dysmorphism, neurological deficits, and infancy death. This was the first time that the *PA1* gene was reported to be associated with Mendelian genetic diseases (Daum et al., 2021). Based on the existing evidence, it is speculated that *PA1* is a potential autosomal recessive causative gene whose homozygous mutation can lead to severe syndromic neurodevelopmental disorders.

## Conclusion

This review summarizes the research into PA1 protein and its biological functions in various processes including its classical H3K4 modification and non-classical function via interacting with transcription factors, as well as those still unknown mechanisms. However, owing to the relatively limited data of the research findings on PA1 functions, a review in this regard needs to be constantly refined. Research could shed more light on the unclarified molecular mechanisms of PA1 in various biological processes and explored its new roles such as in neurodevelopment and tumor development.

## Supplementary Material

Supplementary Figure S1 The expression profile of PA1 in human tissues according to the GeneCards database.

Supplementary Figure S2 The expression of PA1 in different tumor cell lines according to the GeneCards database.
