# Supplementary figures and images for: PTIP-Associated Protein 1: More Than a Component of the MLL3/4 Complex
Source: Front Genet. 2022 Jun 9;13:889109. doi: 10.3389/fgene.2022.889109 (PMC9219552; doi:10.3389/fgene.2022.889109)

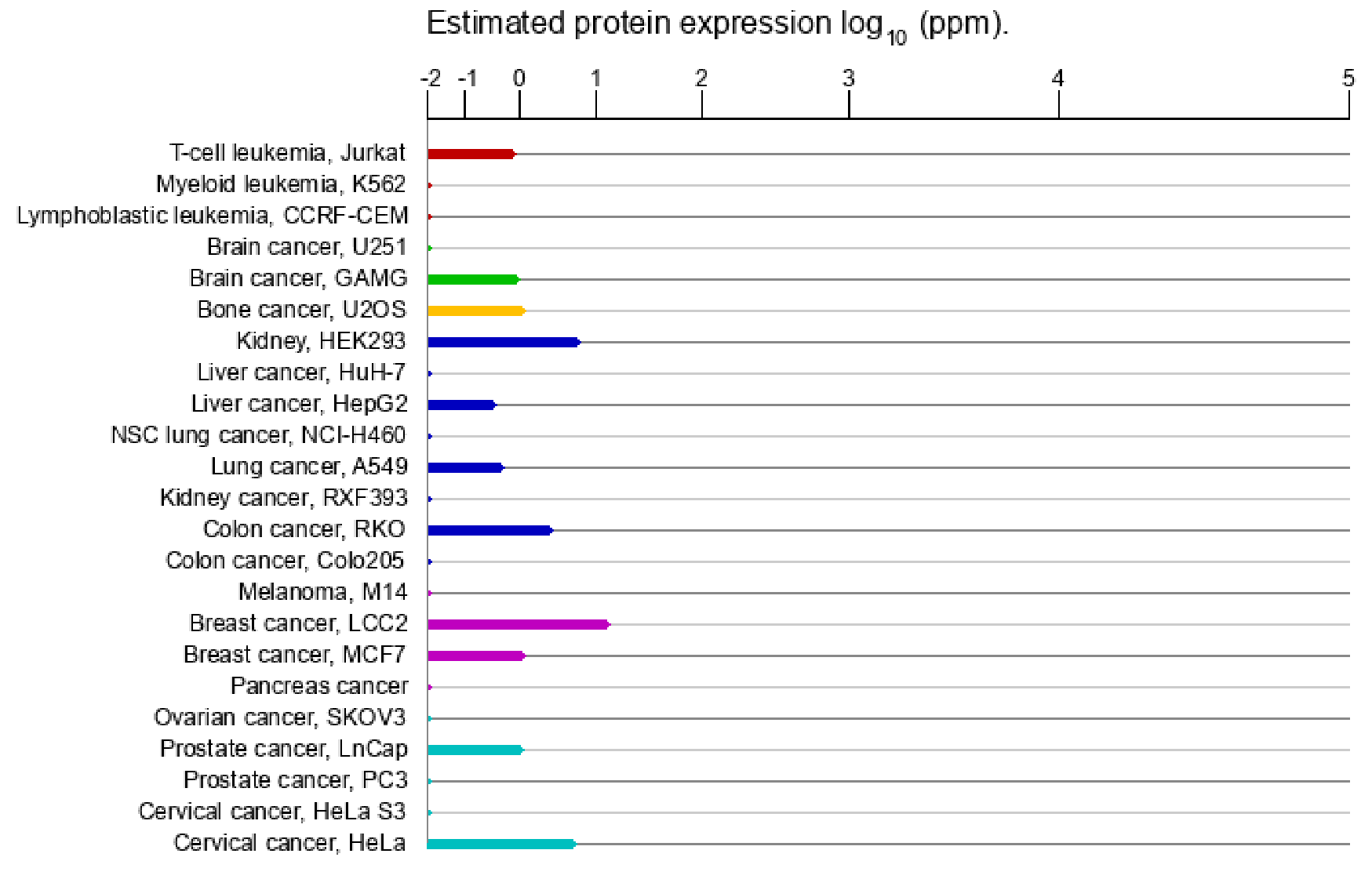

Supplement: Supplementary file 1 [file Image2.TIF]

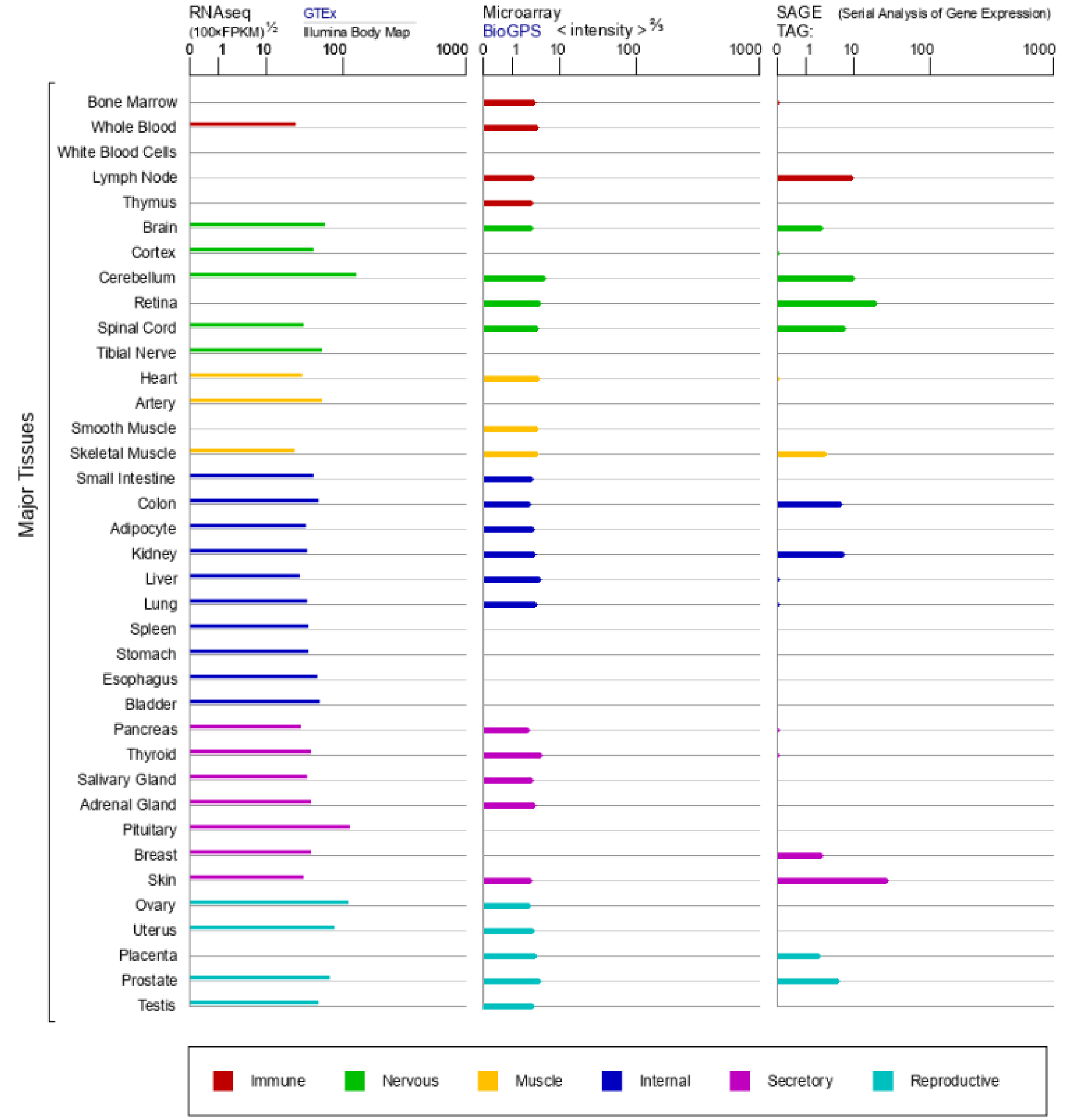

Supplement: Supplementary file 2 [file Image1.TIF]
